# A Global Survey on Diagnostic, Therapeutic and Preventive Strategies in Intensive Care Unit—Acquired Weakness

**DOI:** 10.3390/medicina58081068

**Published:** 2022-08-08

**Authors:** Felix Klawitter, Marie-Christine Oppitz, Nicolai Goettel, Mette M. Berger, Carol Hodgson, Steffen Weber-Carstens, Stefan J. Schaller, Johannes Ehler

**Affiliations:** 1 Department of Anesthesiology and Intensive Care Medicine, University Medical Center Rostock, 18057 Rostock, Germany; 2 Department of Anesthesiology, University of Florida College of Medicine, Gainesville, FL 32610, USA; 3 Department of Clinical Research, University of Basel, 4031 Basel, Switzerland; 4 Service of Adult Intensive Care, Lausanne University Hospital (CHUV), 1011 Lausanne, Switzerland; 5 Australian and New Zealand Intensive Care Research Centre, School of Public Health and Preventive Medicine, Monash University, Melbourne, VIC 3004, Australia; 6 Department of Intensive Care and Hyperbaric Medicine, The Alfred, Melbourne, VIC 3004, Australia; 7 Department of Anesthesiology and Operative Intensive Care Medicine (CCM/CVK), Charité—Universitätsmedizin Berlin, Augustenburger Platz 1, 13357 Berlin, Germany; 8 Department of Anesthesiology and Operative Intensive Care Medicine (CVK/CCM), Charité—Universitätsmedizin Berlin, 10117 Berlin, Germany; 9 Department of Anesthesiology and Intensive Care, Klinikum Rechts der Isar, School of Medicine, Technical University of Munich, 81675 Munich, Germany

**Keywords:** surveys and questionnaires, critical care, neuromuscular diseases, critical illness myopathy, critical illness polyneuropathy

## Abstract

*Background and Objectives:* Intensive care unit-acquired weakness (ICU-AW) is one of the most frequent neuromuscular complications in critically ill patients. We conducted a global survey to evaluate the current practices of diagnostics, treatment and prevention in patients with ICU-AW. *Materials and Methods*: A pre-survey was created with international experts. After revision, the final survey was endorsed by the European Society of Intensive Care Medicine (ESICM) using the online platform SurveyMonkey^®^. In 27 items, we addressed strategies of diagnostics, therapy and prevention. An invitation link was sent by email to all ESICM members. Furthermore, the survey was available on the ESICM homepage. *Results*: A total of 154 healthcare professionals from 39 countries participated in the survey. An ICU-AW screening protocol was used by 20% (28/140) of participants. Forty-four percent (62/141) of all participants reported performing routine screening for ICU-AW, using clinical examination as the method of choice (124/141, 87.9%). Almost 63% (84/134) of the participants reported using current treatment strategies for patients with ICU-AW. The use of treatment and prevention strategies differed between intensivists and non-intensivists regarding the reduction in sedatives (80.0% vs. 52.6%, *p* = 0.002), neuromuscular blocking agents (76.4% vs. 50%, *p* = 0.004), corticosteroids (69.1% vs. 37.2%, *p* < 0.001) and glycemic control regimes (50.9% vs. 23.1%, *p* = 0.002). Mobilization and physical activity are the most frequently reported treatment strategies for ICU-AW (111/134, 82.9%). The availability of physiotherapists (92/134, 68.7%) and the lack of knowledge about ICU-AW within the medical team (83/134, 61.9%) were the main obstacles to the implementation of the strategies. The necessity to develop guidelines for the screening, diagnosing, treatment and prevention of ICU-AW was recognized by 95% (127/133) of participants. *Conclusions*: A great heterogeneity regarding diagnostics, treatment and prevention of ICU-AW was reported internationally. Comprehensive guidelines with evidence-based recommendations for ICU-AW management are needed.

## 1. Introduction

Intensive care unit-acquired weakness (ICU-AW) is one of the most frequent neuromuscular complications associated with prolonged intensive care treatment lasting up to several days, increased morbidity and mortality [1,2]. Furthermore, the quality of life is severely affected even months after hospital discharge in patients with ICU-AW [3]. Hallmark symptoms include a symmetric, flaccid palsy, reduced muscle tone as well as reduced or absent muscle reflexes [4]. By definition, ICU-AW is clinically diagnosed by the determination of typical neurological symptoms combined with a Medical Research Council—sum score (MRC-SS) < 48 [5]. Current evidence demonstrates that an MRC-SS < 55 is associated with relevant neuromuscular dysfunction and results in a poorer patient outcome [6,7]. Many different approaches for diagnostics, treatment and prevention have been published in recent years, but little is known about their implementation and acceptance in daily intensive care practice [4,8,9,10,11,12]. Early mobilization and physiotherapy have been identified as cornerstones in the treatment and prevention of ICU-AW [11,13,14]. However, recent evidence suggests a significant heterogeneity in the regional intensive care medicine of patients with ICU-AW as well as insufficient knowledge about the syndrome itself [15]. Furthermore, evidence-based recommendations for the management of ICU-AW are lacking, potentially resulting in the heterogeneity of medical care in patients with ICU-AW depending on individual expertise and available resources. We therefore conducted an international survey to evaluate the current clinical practice of diagnostics, monitoring, treatment and prevention strategies in patients with ICU-AW.

## 2. Materials and Methods

### 2.1. Questionnaire and Study Participation

**Figure 1:**
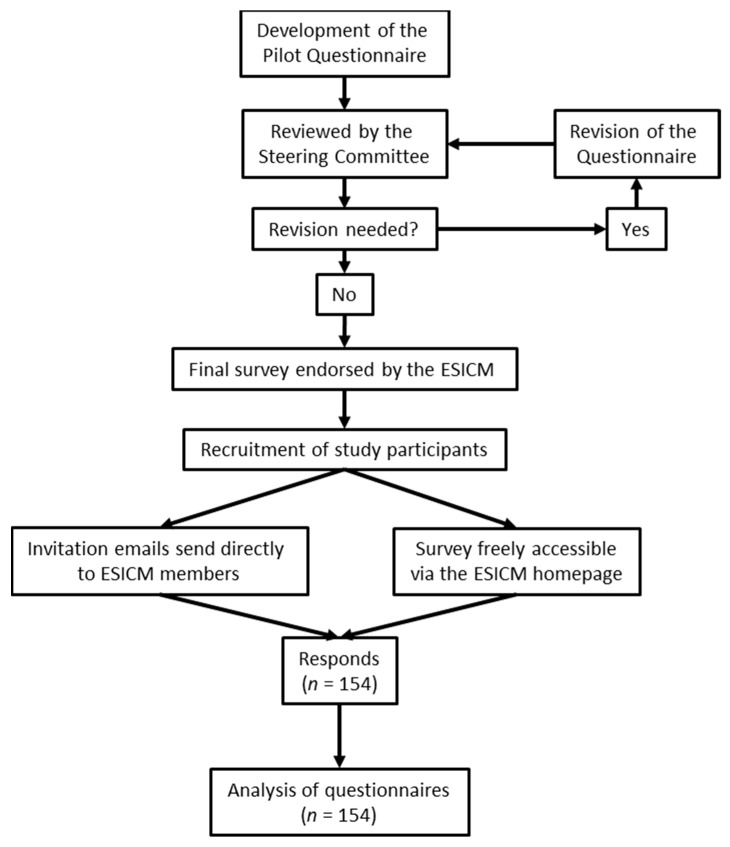
Study flow chart.

We conducted an international cross-sectional online survey between July and November 2021, adhering to the published Consensus-Based Checklist for Reporting of Survey Studies (CROSS) [16]. The survey was endorsed by the European Society of Intensive Care Medicine (ESICM) and approved by the local ethics committee of the University of Rostock (A 2021-0111). Based on current scientific literature, we developed a new questionnaire containing 27 items organized into three categories: (1) “basic demographic data”, (2) “diagnostic and monitoring strategies” and (3) “treatment and prevention strategies” (Figure 1) [7,9,17]. Single and multiple answer questions as well as one open-ended question were included. The first version (V. 1.0) of the questionnaire was reviewed and revised four times (V. 1.1, 1.2, 1.3, 1.4) in total by F.K., J.E. and S.J.S. Members of the steering committee (N.G., C.H., M.M.B. and S.W.C.) who had not drafted the first version pre-tested the questionnaire (V. 1.4) afterwards. Building up on these reviews, we developed the final questionnaire (V. 2.0), which was lastly reviewed and approved by all members of the steering committee (Supplementary File S1). The survey was addressed to all healthcare professionals working with intensive care patients. A sampling of study participants was performed in two simultaneously operating ways: (1) the questionnaire was freely accessible online through the ESICM homepage at SurveyMonkey^®^ (Momentive Inc., San Mateo, CA, USA) and (2) an email containing a link to the survey was sent out by the ESICM inviting all registered members to participate in the study. Furthermore, the ESICM re-sent two invitation emails calling for study participation in August and October 2021. Study participation was voluntary and no prior registration was necessary for study participation. Within the survey, no personal data were collected and backtracking of study participants was not possible. The survey data were collected on external password-secured servers and access was granted only to members of the steering committee.

### 2.2. Statistical Analysis

For statistical analysis, we used MS-Excel 2010 (Microsoft, Redmond, WA, USA) and IBM SPSS Statistics (Version 25, IBM Corp., Armonk, NY, USA). Data are presented as sum (percent) or mean (standard deviation). Chi-square test with Yates correction was used for all categorical variables. In case of expected values of <5 in the 2 × 2 contingency table, we used the Fisher’s exact test. Statistical significance was indicated by a *p* value < 0.05.

## 3. Results

### 3.1. Part One: Basic Demographic Data

In total, we received 154 questionnaires from 146 different ICUs in 39 countries around the world (the distribution of participating countries is listed in Supplementary File S2). The basic demographic data are provided in Table 1. Not all questions were answered by all participants, as indicated by different absolute counts. The most frequent primary medical specialties of the participants included intensivists (study participants with the primary medical specialty ‘Intensive Care Medicine’; 64/153, 41.8%) and non-intensivists (89/153, 58.2%, including study participants with the primary medical specialty ‘Physiotherapy’ (30/89, 33.7%), ‘Anesthesiology’ (29/89, 32.6%) and others, such as ‘Nursing’, ‘Neurology’, ‘Internal Medicine and Surgery’ (all together 30/89, 33.7%)). Seventy-seven percent (110/142) of all participating healthcare professionals worked as consultant/medical specialist or as Chief/Head of Department. Almost two-thirds of all study participants (96/152, 63.2%) practiced for more than 10 years in their profession. The number of differently sized ICUs was almost equally distributed. The majority (120/153, 78.4%) were interdisciplinary (medical and surgical) ICUs. About sixty-three percent (95/152) of all study participants worked on an ICU at a university hospital. About two-thirds of the study participants (103/152, 67.8%) reported neuromuscular complications, including ICU-AW, to be a relevant research topic.

### 3.2. Part Two: Diagnostic and Monitoring Strategies

A standardized protocol for the screening and detection of patients with ICU-AW was reported to be used by 20% of study participants (28/140). In particular, clinical examination (124/141, 87.9%) as well as selective scores (46/141, 32.6%) and electrophysiological methods (46/141, 32.6%), such as electroneurography (ENG) and electromyography (EMG), were reported as the most frequently used diagnostic tests (Table 2). We found no significant differences between university and non-university hospitals or intensivists and non-intensivists regarding the screening strategy. Furthermore, most clinicians reported that they started screening for ICU-AW in patients with higher disease severity assuming a higher ICU-AW probability (72/141, 51.1%). Routine screening for ICU-AW independently from disease severity was declared to be initiated by 44% (62/141) of all study participants. Physicians were, among the different groups of healthcare professionals, the most likely to perform the initial screening (73/141, 51.8%). This was confirmed by 70.9% (100/141) of participants who reported that physicians performed the daily screening at their ICU. Fifty-six percent (80/141) of participants performed screening tests for the presence of ICU-AW once a day. After the detection of ICU-AW, the preferred following method to verify the diagnosis was ENG/EMG (63/140, 45.0%). According to 35.7% (50/140) of responders, a neurologist was consulted to confirm the diagnosis of ICU-AW. In 27.9% (39/140) of responders, no further diagnostic was applied. The majority (70/141, 49.6%) of participants did not use scores for the assessment of physical deficits in ICU patients. The modified Rankin scale (mRS) was, if scores were performed in the ICU, the most frequently reported score to assess the functional disability.

### 3.3. Part Three: Treatment and Prevention Strategies

**Figure 2:**
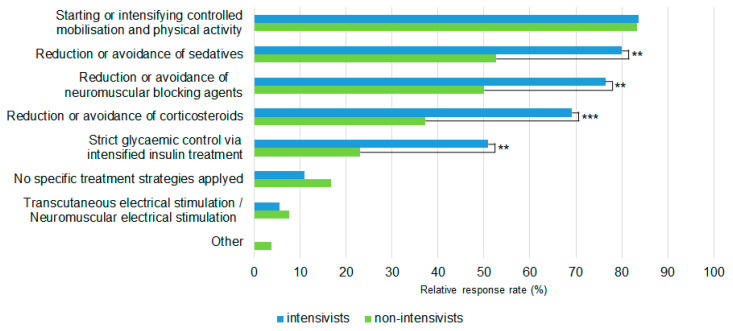
Treatment and prevention strategies between intensivists (primary specialty) and non-intensivists. **: *p* < 0.01. ***: *p* < 0.001.

In total, 62.7% (84/134) of all study participants reported the availability of specific treatment strategies for ICU-AW within their ICU. In particular, the most frequently applied strategies were the beginning or intensifying of mobilization and physical activity (111/134, 82.8%), the reduction or avoidance of sedatives (85/134, 63.4%) and the reduction or avoidance of neuromuscular blocking agents (81/134, 60.4%) (Table 3). We found no significant differences between university and non-university hospitals in terms of mobilization and physical activity. However, intensivists and non-intensivists significantly differed in their statements about applied treatment and prevention strategies regarding the reduction in sedatives (80.0% vs. 52.6%, *p* = 0.002), neuromuscular blocking agents (76.4% vs. 50%, *p* = 0.004), corticosteroids (69.1% vs. 37.2%, *p* < 0.001) and strict glycemic control regimes (50.9% vs. 23.1%, *p* = 0.002, Figure 2). In more than half of all cases, physiotherapy and mobilization was reported to be performed once a day (73/134, 54.5%), and in 32.8% (44/134) of cases, multiple times per day. The three main reported barriers to the treatment of ICU-AW patients were: (1) the availability of physiotherapists (92/134, 68.7%), (2) the lack of knowledge about ICU-AW among the medical staff (83/134, 61.9%) and (3) the availability of diagnostic/therapeutic approaches (81/134, 60.4%). We found no significant differences between university and non-university hospitals or intensivists and non-intensivists in their opinion about deficits in medical care. Most participants (100/134, 74.6%) reported that the family members of patients with ICU-AW were informed about possible long-term physical disability, 22.4% (30/134) were not informed and 3% (4/134) were not certain whether family members were informed about possible long-term consequences of ICU-AW. The diagnosis of ICU-AW was reported to be listed in the medical history of patients by 65.7% (88/134) of responders compared to 30.6% (41/134) of responders, where the ICU-AW was not listed.

Forty-seven percent (63/134) of participants reported that their patients with ICU-AW were transferred to a neurological rehabilitation center after hospital discharge, but 48.5% (65/134) did not. The great majority (127/133, 95.5%) of all study participants supported the development of evidence-based guidelines for the diagnosis, monitoring, treatment and prevention of ICU-AW to improve patient care.

## 4. Discussion

The present survey was the first worldwide approach to systemically depict the current global state of care in patients with ICU-AW. The results from this survey represent different levels of experience from a broad spectrum of healthcare professionals with short- and long-term clinical practices in intensive care medicine.

Our results indicate a great heterogeneity in the screening, diagnostic, treatment and prevention strategies worldwide, as well as the presence of multiple barriers against the implementation of strategies to mitigate ICU-AW. A well-structured concept for the management of ICU-AW was implemented in a minority of ICUs.

### 4.1. Diagnostic and Monitoring Strategies

Only 20% of participants stated that a standardized approach to screen for and diagnose ICU-AW was implemented at their ICU, suggesting significant barriers in clinical practice. According to our data, the clinical examination was the preferred screening method for ICU-AW. Only one-third of participants used the MRC-SS for the screening of ICU-AW. Recently, Van Aerde et al. reported on a correlation between even subtle changes detected with the MRC-SS and patient outcome, which underlines the importance of using validated scores such as the MRC-SS in the clinical setting [6]. However, a certain level of patient compliance is mandatory for a reliable clinical examination, including muscle strength assessment, which is often difficult to perform in the early stages of critical illness due to the sedation and mechanical ventilation [18]. To date, compliance-independent measures of muscle force remain experimental and need further validation before a broad implementation in daily clinical practice [19]. Furthermore, recent emerging methods such as selective risk scores, body fluid biomarkers, simplified electroneurography or neuromuscular ultrasound are interesting new attempts in the screening for ICU-AW, especially in the context of a compliance-independent assessment [8,20,21,22,23]. However, according to our study, they are only partially used in clinical practice. A possible explanation could be the lack of randomized validation studies or a missing correlation with patient outcome within the methods mentioned above. The early detection of physical disability in ICU patients is assumed to be an important cornerstone for the initiation of treatment strategies, as suggested by studies comparing the effect of early versus late mobilization [14]. This is in accordance with our data, where screening for ICU-AW would be initiated mostly in patients with a corresponding risk profile, even before the occurrence of symptoms.

To differentiate the cause of the neuromuscular dysfunction, further electrophysiologic diagnostics are recommended after diagnosing ICU-AW [5]. In our study, only 45% of participants used ENG or EMG, one-third consulted a neurologist and nearly 28 percent did not use any further diagnostics. A distinction between these pathological entities seems reasonable, since it could provide prognostic information on the clinical course of physical disability and the rehabilitation success [24]. Other methods such as muscle/nerve biopsies, body fluid biomarkers or neuromuscular imaging seem negligible in further diagnostics after detecting ICU-AW, perhaps due to their invasiveness, costs and inability to differentiate nervous and muscular damage [25].

Scoring systems assessing the physical function of critically ill patients could help to objectify the extent of functional disability and to monitor the success of mobilization and physiotherapy [26]. However, according to our results, they are not widely used in current clinical practice to monitor patients with ICU-AW. This may be due to the fact that many of these detailed scores appear impracticable and time consuming in daily practice or mostly depend on patient cooperation, which can be difficult in the presence of sedation or prolonged cognitive impairment.

### 4.2. Treatment and Prevention Strategies

Only two-thirds of all participating ICUs applied specific treatment and prevention strategies for critically ill patients at risk for ICU-AW. With the exception of mobilization and physiotherapy, none of the other treatment and prevention strategies were broadly established in daily practice. Physiotherapy and mobilization have been shown to improve muscle strength and may improve functional patient outcome [11]. The time frame for starting physical treatment also seems to play an important role. Recent evidence describes a potential benefit of early mobilization to prevent and improve outcomes in ICU-AW, especially when started within the first 48–72 h [13,27,28,29]. Furthermore, early mobilization has been shown to be safe even in patients with a reduced level of consciousness [16]. According to the present survey, the physiotherapy or mobilization of patients with ICU-AW was reported to occur mostly once a day. Current evidence indicates an impact of the dosage of mobilization on patient outcome, i.e., intensified physiotherapy and mobilization regimes would be desirable in intensive care medicine [30,31].

Furthermore, a subgroup analysis of the different medical specialties revealed that a reported reduction or avoidance of sedatives, neuromuscular blocking agents and corticosteroids as well as glycemic control were significantly less often performed by non-intensivists compared to intensivists, suggesting an influence of the primary medical specialty on the strategies or the relevance and attention attributed to the ICU-AW. There is strong evidence that extensive sedation leads to prolonged immobilization, invasive ventilation and a higher morbidity and mortality [32,33,34]. The impact of sedatives as a risk factor for ICU-AW was recently highlighted [35]. Therefore, national and international guidelines recommend the critical evaluation and reduction of sedation to a needed minimum [36,37].

Hyperglycemia has been considered as another risk factor for ICU-AW [9,38,39]. However, the quality of evidence suggesting a positive effect of strict glycemic control on the prevention of neuromuscular complications remains limited with only a few studies evaluating this topic [40,41,42].

The effects of neuromuscular blocking agents (NMBAs) on the development of ICU-AW remain controversial. Price et al. reviewed numerous articles, concluding a moderate association with ICU-AW, but pointed out a possible reporting bias in many of these studies [43]. A meta-analysis by Yang et al. identified NMBA as a clear trigger factor for ICU-AW in a multiple regression analysis, whereas recent evidence suggested an increased risk only for the development of critical illness polyneuropathy in septic shock patients [44,45]. Lyu et al. highlighted no clear association between NMBA and ICU-AW [46].

The impact of corticosteroid use on the development of ICU-AW is also controversially discussed in the literature. Hermans et al. reviewed prospective studies of mixed quality indicating a more complex interplay of corticosteroids and muscle damage related to the dose and the time period of drug administration [47]. In contrast, a recent meta-analysis suggested that corticosteroid use is strongly associated with clinically detectable muscle weakness, but not with electrophysiological changes [44]. Therefore, current evidence seems too heterogeneous to give a clear statement.

### 4.3. Barriers and Deficits

Based on the present survey, the main reported barriers and deficits associated with ICU-AW management were the lack of available physiotherapists and diagnostic/therapeutic approaches. In the context of the increasing numbers of ICU patients and shortages of medical staff, it is comprehensible that more staff resources are needed to provide adequate intensive care. This is supported by Penoyer et al., showing a correlation between intensive care outcome and the availability of nursing staff [48]. The implementation of specific prevention and treatment strategies such as early mobilization programs and intensified physiotherapy seems evidently only practicable with an increase in trained medical staff. This may inevitably come with increased costs in personal and material resources and to date, to our best knowledge, no sufficient data are available on the cost-effectiveness of early rehabilitation programs in patients with ICU-AW. Future studies evaluating this important issue are desirable. However, from a medical perspective and supported by the strong evidence listed above, early rehabilitation efforts seem clearly beneficial to improve patient outcome and quality of life.

Congruent with recent evidence, our data indicate that a lack of knowledge about ICU-AW among the medical staff is one of the major issues in clinical practice [15]. Failure to address muscle weakness in the ICU is concerning, considering that the higher associated mortality and the negative long-term effects lasting up to 5 years after the ICU remain, independently of the underlying disease [6,7,49,50]. On the other hand, it was positively surprising that in most ICUs worldwide, the diagnosis of ICU-AW was reported to be listed in the medical history; furthermore, family members were informed about possible long-term physical disability due to ICU-AW. Appropriate informational exchange with the family of the critically ill patients has been shown to strengthen social backup and resilience factors regarding the burden of critical illness [51].

It is noteworthy that the above-mentioned issues presented in our study as well as other potential barriers, such as inconsistent nomenclature, lack of facilities and equipment or a failed implementation of preventive strategies have already been identified about ten years ago [52]. This underlines the need for appropriate educational programs, sophisticating human resources management and high-quality evidence to improve medical care for critically ill patients who developed or are at risk of developing ICU-AW.

### 4.4. Strengths and Limitations

The present study assembles the current practices in the diagnosis, monitoring, treatment and prevention of ICU-AW from a broad basis of different ICUs and countries around the world, with results not limited by certain geographic areas. We included different medical professionals (physicians, physiotherapists, nurses), which together with the broad geographical representation, increased the generalizability of our results. Within the study, we adhered to the published criteria for reporting surveys and pre-tested the questionnaire [17].

Some limitations of the study need discussion. First, the relatively small number of responding ICUs limits the generalizability of the survey. Because the survey access was freely available online, we cannot provide a response rate. Due to the small number of participating ICUs in some countries, the results here may not fully depict the current state of the art. Otherwise, there was a lack of information regarding diagnostic and therapeutic attempts for the management of ICU-AW, which makes the results of the present study valuable. Confirmation in appropriate observational studies is nevertheless warranted. The reported results within the present survey need to be confirmed in future observational studies. Second, we did not cover in detail the full range of possible issues associated with ICU-AW, such as associated dysphagia or ventilator-induced diaphragmatic dysfunction. For the sake of practicability, we tried to include the most essential core topics of this severe complication in an easy and rapidly answerable questionnaire. Third, due to technical aspects, we were not able to fully exclude duplicated answers.

## 5. Conclusions

The present study reported current daily practices in the management of ICU-AW. Screening, diagnostic tests, treatment and prevention strategies for ICU-AW are heterogeneous and not standardized, although long-term consequences are evident. Comprehensive guidelines with evidence-based recommendations are needed and recommended by most healthcare professionals for the implementation of structured approaches to ICU-AW.

## Figures and Tables

**Table 1:** Basic demographic data. Absolute counts indicate all participants who chose the particular answer of the corresponding question. Total counts indicate all study participants who answered the question at all. Relative counts indicate the ratio of absolute to total counts in percentages.

**Primary medical specialty (SC)**	**Absolute/Total (*n*/153)**	**Relative (%)**
(a) Intensive Care Medicine	64	41.8
(b) Anesthesiology	29	19.0
(c) Internal Medicine	16	10.5
(d) Surgery	1	0.7
(e) Neurology	2	1.3
(f) Nursing	4	2.6
(g) Physiotherapy	30	19.6
(h) Other	7	4.6
**Medical training status (SC)**	**Absolute/Total (*n*/142)**	**Relative (%)**
(a) Resident/in-training	10	7.0
(b) Fellow/training completed	22	15.5
(c) Consultant/medical specialist	77	54.2
(d) Chief/Head of Department	33	23.2
**Years of intensive care practice (SC)**	**Absolute/Total (*n*/152)**	**Relative (%)**
(a) <5	18	11.8
(b) 5–10	38	25.0
(c) 11–15	25	16.4
(d) 16–20	27	17.8
(e) >20	44	28.9
**Type of hospital (SC)**	**Absolute/Total (*n*/152)**	**Relative (%)**
(a) University hospital	95	62.5
(b) Non-university hospital	57	37.5
**Number of ICU beds (SC)**	**Absolute/Total (*n*/153)**	**Relative (%)**
(a) <10	10	6.5
(b) 10–20	49	32.0
(c) 21–50	51	33.3
(d) >50	43	28.1
**Type of ICU specialty (MC)**	**Absolute/Total (*n*/153)**	**Relative (%)**
(a) Perioperative (surgical/anesthesiological)	15	9.8
(b) Internal medicine	11	7.2
(c) Neurologic ICU	5	3.3
(d) Pediatric ICU	2	1.3
(e) Interdisciplinary (medical and surgical) ICU	120	78.4
**Is research on ICU-AW a topic for you? (SC)**	**Absolute/Total (*n*/152)**	**Relative (%)**
YES	103	67.8
NO	45	29.6
I don’t know	4	2.6

ICU: intensive care unit. ICU-AW: intensive care unit-acquired weakness. MC: multiple-choice question. SC: single-choice questions.

**Table 2:** Screening, diagnostics and monitoring of ICU-AW. Absolute counts indicate all participants who chose the particular answer of the corresponding question. Total counts indicate all study participants who answered the question at all. Relative counts indicate the ratio of absolute to total counts in percent.

**Is a standard I** **in-house protocol used for the screening of ICU-AW? (SC)**	**Absolute/Total (*n*/140)**	**Relative (%)**
(a) Yes	28	20.0
(b) No	112	80.0
(c) I don’t know	0	0
**Routinely used screening methods (MC)**	**Absolute/Total (*n*/141)**	**Relative (%)**
(a) Clinical examination	124	87.9
(b) Selective scores (e.g., Medical Research Council—sum score, MRC-SS)	46	32.6
(c) Electrophysiology (electroneurography/electromyography)	46	32.6
(d) Neuromuscular ultrasound	10	7.1
(e) Laboratory diagnostics including body fluid biomarkers	8	5.7
(f) Muscle/nerve biopsy	6	4.3
(g) No screening is performed	18	12.8
(h) I do not know	4	2.8
(i) Other	2	1.4
**Most likely circumstances of screening for ICU-AW (MC)**	**Absolute/Total (*n*/141)**	**Relative (%)**
(a) Routinely, within the daily clinical examinations	62	44.0
(b) Occasionally, when ICU-AW seems likely according to disease severity and clinical course	72	51.1
(c) Occasionally, when my patient shows no spontaneous limb movements or inadequate motoric responses over a period of time	50	35.5
(d) Occasionally, after the first failed weaning from the respirator	23	16.3
(e) Screening for ICU-AW is not performed	6	4.3
(f) Other	4	2.8
**Who should primarily screen? (SC)**	**Absolute/Total (*n*/141)**	**Relative (%)**
(a) Physicians	73	51.8
(b) Nurses	18	12.8
(c) Physiotherapists	46	32.6
(d) I don’t know	4	2.8
**Who is screening? (MC)**	**Absolute/Total (*n*/141)**	**Relative (%)**
(a) Physicians	100	70.9
(b) Nurses	38	27.0
(c) Physiotherapists	62	44.0
(d) I don’t know	7	5.0
**Screening intervals used (SC)**	**Absolute/Total (*n*/141)**	**Relative (%)**
(a) Once per patient stay	13	9.2
(b) Once daily	80	56.7
(c) Once per ICU shift	9	6.4
(d) None of the above mentioned	29	20.6
(e) Never	10	7.1
**Diagnostics after detection of ICU-AW (MC)**	**Absolute/Total (*n*/140)**	**Relative (%)**
(a) Electrophysiology (electroneurography/electromyography)	63	45.0
(b) Neuromuscular ultrasound	9	6.4
(c) Muscle/nerve biopsy	5	3.6
(d) Consultation by an expert neurologist	50	35.7
(e) Laboratory diagnostics including body fluid biomarkers	11	7.9
(f) Further diagnostic is not performed	39	27.9
(g) I don’t know	9	6.4
(h) Other	1	0.7
**Functional disability scores (MC)**	**Absolute/Total (*n*/141)**	**Relative (%)**
(a) Modified Rankin scale (mRS)	32	22.7
(b) Barthel Index (BI)	23	16.3
(c) Functional independence measure (FIM)	6	4.3
(d) Physical function in the ICU test (PFIT)	6	4.3
(e) Functional status score for ICU (FSS-ICU)	8	5.7
(f) Acute Care Index of Function (ACIF)	4	2.8
(g) Scores are not used	70	49.6
(h) I don’t know	9	6.4
(i) Other	14	9.9

ICU: intensive care unit. ICU-AW: intensive care unit-acquired weakness. MC: multiple-choice question. SC: single-choice question.

**Table 3:** Treatment and prevention strategies. Absolute counts indicate all participants who chose the particular answer of the corresponding question. Total counts indicate all study participants who answered the question at all. Relative counts indicate the ratio of absolute to total counts in percentages.

**Are treatment strategies available at your ICU? (SC)**	**Absolute/Total (*n*/134)**	**Relative (%)**
(a) Yes	84	62.7
(b) No	44	32.8
(c) I don’t know	6	4.5
**What specific treatment/prevention strategies do you use? (MC)**	**Absolute/Total (*n*/134)**	**Relative (%)**
(a) Starting or intensifying controlled mobilization and physical activity	111	82.8
(b) Transcutaneous electrical stimulation (TENS)/neuromuscular electrical stimulation	9	6.7
(c) Strict glycaemic control via intensified insulin treatment	46	34.3
(d) Reduction or avoidance of neuromuscular blocking agents	81	60.4
(e) Reduction or avoidance of corticosteroids	67	50.0
(f) Reduction or avoidance of sedatives	85	63.4
(g) We apply no specific treatment strategies after diagnosing ICU-AW	19	14.2
(h) Other	3	2.2
**Specify the frequency of physiotherapeutic treatment at your ICU. (SC)**	**Absolute/Total (*n*/134)**	**Relative (%)**
(a) Once a day	73	54.5
(b) Once a working shift	23	17.2
(c) Multiple times per working shift	21	15.7
(d) No regular intervals	14	10.4
(e) Never/none	3	2.2
**Which deficits in regard to medical care of ICU-AW patients exist? (MC)**	**Absolute/Total (*n*/134)**	**Relative (%)**
(a) Availability of diagnostic/therapeutic approaches	81	60.4
(b) Not enough physiotherapists available	92	68.7
(c) Not enough nurses available	42	31.3
(d) Not enough physicians available	12	9.0
(e) Not enough knowledge about ICU-AW within medical staff	83	61.9
(f) Not enough time to care about patients with ICU-AW within the medical staff	54	40.3
(g) There are no deficits	1	0.7
(h) I don’t know	2	1.5
(i) Other	6	4.5

ICU: intensive care unit. ICU-AW: intensive care unit-acquired weakness. MC: multiple-choice question. SC: single-choice question.

## Data Availability

The dataset used and/or analyzed within the present study is available from the corresponding author upon reasonable request.

## Supplementary Materials

The following supporting information can be downloaded at: https://www.mdpi.com/article/10.3390/medicina58081068/s1, Supplementary File S1: Survey questionnaire. Supplementary File S2: Table S1: Listing of participating countries within the survey.
