# Supplementary material for: A Global Survey on Diagnostic, Therapeutic and Preventive Strategies in Intensive Care Unit—Acquired Weakness
Source: Medicina (Kaunas). 2022 Aug 8;58(8):1068. doi: 10.3390/medicina58081068 (PMC9416039; doi:10.3390/medicina58081068)
Supplement: Supplementary file 1 [file medicina-58-01068-s001.zip › medicina-1832858-supplementary-File S1.pdf]

The Intensive Connection

Diagnostic, monitoring and rehabilitation strategies in patients with Intensive Care Unit – Acquired Weakness – a survey on European intensive care units

## 1. Survey participation

1.1 We are conducting this online-survey to assess the current practice of diagnostic, monitoring and rehabilitation strategies in patients with Intensive Care Unit – Acquired Weakness (ICU-AW) at intensive care units across Europe. ICU-AW is one of the most important neuromuscular complications in critically ill patients and is associated with increased morbidity and mortality. Participation in this online-survey is voluntary and anonymous. If you enter the name of the unit it will be replaced by a code afterwards and the ICU name removed. This survey contains 27 questions and no more than ten minutes are necessary to complete this questionnaire.

Thank you very much for your participation!

Kind regards,

Johannes Ehler, MD

Felix Klawitter, MD

Stefan Schaller, MD

## 2. Basic demographic data

### 2.1 Please select your country:

…

### 2.3 Please provide the abbreviated name of your ICU (like M101i or ICU1) – We will replace this name with an arbitrary number.

…

### 2.4 Please specify your primary medical speciality.

a) Intensive Care Medicine

b) Anaesthesiology

c) Internal Medicine

d) Surgery

e) Neurology

f) Nursing

g) Physiotherapy

h) If other, please specify:…

**2.5 Please specify your medical training status (select the answer that fits best to your current position).**

a) Resident / In-Training

b) Fellow / training completed

c) Consultant / medical specialist

d) Chief / Head of Department

**2.6 How many years do you practice intensive care medicine in total?**

a) <5

b) 5-10

c) 11-15

d) 16-20

e) >20

**2.7 Please specify the current hospital you are working at**

a) University hospital

b) Non-university hospital

**2.8 Please specify the total number of intensive care beds at your hospital.**

a) <10

b) 10-20

c) 21-50

d) >50

**2.9 Please specify your intensive care unit (ICU) according to the type of medical specialty.** *(Multiple choice question – please select* ***__all applicable__*** *answers)*

a) Perioperative (surgical / anesthesiological) ICU

b) Internal Medicine ICU

c) Neurologic ICU

d) Paediatric ICU

e) Interdisciplinary (medical and surgical) ICU

**2.10 Is research on ICU-AW or neuromuscular complications in general a relevant topic at your intensive care unit?**

a) YES

b) NO

c) I don’t know

## 3. Diagnostic and monitoring strategies

**3.1 Do you use a standardized protocol (e.g., a standard operating procedure – SOP) for screening and detection of ICU-AW at your intensive care unit?**

a) YES

b) NO

c) I don’t know

**3.2 What methods do you routinely use for systemic screening and detection of ICU-AW at your intensive care unit?** *(Multiple choice question – please select* ***__all applicable__*** *answers)*

a) Clinical examination

b) Selective scores (e.g. Medical Research Council – sum score, MRC-SS)

c) Electrophysiology (electroneurography/electromyography)

d) Neuromuscular ultrasound

e) Laboratory diagnostics including body fluid biomarkers

f) Muscle/Nerve biopsy

g) A screening is not performed

h) I don’t know

i) If other, please specify:…

**3.3 Please specify the MOST LIKELY circumstances you start a screening for ICU-AW.** *(Multiple choice question – please select **all applicable** answers)*

a) Routinely, within the daily clinical examinations

b) Occasionally, when ICU-AW seems likely according to disease severity and clinical course

c) Occasionally, when my patient shows no spontaneous limb movements or inadequate motoric responses over a period of time

d) Occasionally, after the first failed weaning from the respirator

e) A screening for ICU-AW is not performed

f) If other, please specify:…

**3.4 Who should primarily screen the patients for the presence of ICU-AW in your opinion?**

a) Physicians

b) Nurses

c) Physiotherapists

d) I don’t know

**3.5 Who currently screens the ICU patients for the presence of ICU-AW at your intensive care unit?** *(Multiple choice question – please select **all applicable** answers)*

a) Physicians

b) Nurses

c) Physiotherapists

d) I don’t know

**3.6 Which are the intervals you screen your assessable patients for the presence of ICU-AW?**

a) Once per patient stay

b) Once daily

c) Once per ICU shift (e.g. every eight hours)

d) None of the above

e) Never

**3.7 Which of the following diagnostics do you perform after the detection of ICU-AW?)** *(Multiple choice question – please select **all applicable** answers)*

a) Electrophysiology (electroneurography/electromyography)

b) Neuromuscular ultrasound

c) Muscle/Nerve biopsy

d) Consultation of an expert neurologist

e) Laboratory diagnostics including body fluid biomarkers

f) Further diagnostic is not performed

g) I don't know

h) If other, please specify:…

**3.8 Which of the following scores do you use to assess the functional disability of your intensive care patients?** *(Multiple choice question – please select **all applicable** answers)*

a) Modified Rankin Scale (mRS)

b) Barthel Index (BI)

c) Functional Independence Measure (FIM)

d) Physical Function in the ICU Test (PFIT)

e) Functional Status Score for ICU (FSS-ICU)

f) Acute Care Index of Function (ACIF)

g ) Scores are not used

h) I don't know

i ) If other, please specify:…

## 4. Treatment and prevention strategies

**4.1 Do you have a strategy at your ICU for the further intensive care treatment following the diagnosis of ICU-AW (e.g. strict glycaemic control, intensified mobilisation or adjusted weaning strategies)?**

a) YES

b) NO

c) I don’t know

**4.2 Please specify the applied intensive care treatment strategies at your ICU after diagnosing ICU-AW.** *(Multiple choice question – please select* ***all applicable*** *answers)*

a) Starting or intensifying controlled mobilisation and physical activity

b) Transcutaneous electrical stimulation (TENS)/Neuromuscular electrical stimulation

c) Strict glycaemic control via intensified insulin treatment

d) Reduction or avoidance of neuromuscular blocking agents

e) Reduction or avoidance of corticosteroids

f) Reduction or avoidance of sedatives

g) We apply no specific treatment strategies after diagnosing ICU-AW

h) If other, please specify:…

**4.3 How frequently are physiotherapy and controlled mobilisation of patients diagnosed with ICU-AW performed at your ICU?**

a) Once a day

b) Once a working shift

c) Multiple times per working shift

d) No regular intervals

e) None

**4.4 In your opinion, which deficits currently exist in the medical care of patients with ICU-AW?** *(Multiple choice question – please select* ***all applicable*** *answers)*

a) Availability of diagnostic/therapeutic approaches

b) Not enough physiotherapists available

c) Not enough nurses available

d) Not enough physicians available

e) Not enough knowledge about ICU-AW within medical staff

f) Not enough time to care about patients with ICU-AW within the medical staff

g) There are no deficits

h) I don’t know

i) If other, please specify:…

**4.5 Do you routinely inform family members about possible long-term physical disability in patients with ICU-AW?**

a) YES

b) NO

c) I don’t know

**4.6 Do you routinely list the diagnosis of ICU-AW in the medical history of your patients?**

a) YES

b) NO

c) I don’t know

4.7 **Do you regularly transfer patients with ICU-AW to a neurologic rehabilitation center following the ICU stay?**

a) YES

b) NO

c) I don’t know

**4.8 Do you think the development of an evidence-based guideline for the diagnosis, monitoring, treatment and prevention of ICU-AW is meaningful?**

a) YES

b) NO

c) I don’t know

**4.9 Do you have any final comments on the survey?**
