# Supplementary material for: A Global Survey on Diagnostic, Therapeutic and Preventive Strategies in Intensive Care Unit—Acquired Weakness
Source: Medicina (Kaunas). 2022 Aug 8;58(8):1068. doi: 10.3390/medicina58081068 (PMC9416039; doi:10.3390/medicina58081068)
Supplement: Supplementary file 1 [file medicina-58-01068-s001.zip › medicina-1832858-supplementary-S2.pdf]

**Table S1: Participating countries.**

| Countries | Absolute values | Relative values (%) |
|---|---|---|
| Australia | 18 | 11.8 |
| Spain | 15 | 9.8 |
| Germany | 14 | 9.2 |
| United Kingdom | 13 | 8.5 |
| India | 10 | 6.5 |
| Italy | 8 | 5.2 |
| Switzerland | 7 | 4.6 |
| France | 6 | 3.9 |
| Portugal | 6 | 3.9 |
| United States of America | 5 | 3.3 |
| Brazil | 5 | 3.3 |
| New Zealand | 4 | 2.6 |
| Poland | 3 | 2.0 |
| Austria | 3 | 2.0 |
| Greece | 3 | 2.0 |
| Mexico | 3 | 2.0 |
| Russia | 2 | 1.3 |
| Finland | 2 | 1.3 |
| Ireland | 2 | 1.3 |
| Sweden | 2 | 1.3 |
| Romania | 2 | 1.3 |
| Canada | 2 | 1.3 |
| Turkey | 2 | 1.3 |
| Uruguay | 1 | 0.7 |
| Ecuador | 1 | 0.7 |
| Iceland | 1 | 0.7 |
| Sudan | 1 | 0.7 |
| Libya | 1 | 0.7 |
| Lebanon | 1 | 0.7 |
| Bahrain | 1 | 0.7 |
| Indonesia | 1 | 0.7 |
| Japan | 1 | 0.7 |
| Singapore | 1 | 0.7 |
| South Africa | 1 | 0.7 |
| Saudi Arabia | 1 | 0.7 |
| Netherlands | 1 | 0.7 |
| Israel | 1 | 0.7 |
| Georgia | 1 | 0.7 |
| Egypt | 1 | 0.7 |
